# Disseminated varicella-zoster virus infection in an aplastic anemia- paroxysmal nocturnal hemoglobinuria syndrome patient: A case report

**DOI:** 10.3389/fcimb.2023.1163872

**Published:** 2023-04-19

**Authors:** Jie Wang, Zheng Yang, Danhong Ren, Zhanli Shi, Kun Fang, Zhihui Li

**Affiliations:** Department of Intensive Care Unit, Hangzhou Red-Cross Hospital, Hangzhou, Zhejiang, China

**Keywords:** disseminated varicella zoster infection, varicella zoster virus, aplastic anemia, AA-PNH syndrome, case report

## Abstract

**Background:**

Varicella-zoster virus (VZV) is a common and widespread human-restricted pathogen. It is famous for its dermatological manifestations, such as varicella and herpes zoster. Patients with aplastic anemia-paroxysmal nocturnal hemoglobinuria (AA-PNH) syndrome complicated with fatal disseminated varicella zoster virus infection are very rare and in danger.

**Patient concerns:**

A 26-year-old man with a history of AA-PNH syndrome was receiving cyclosporine and corticosteroid treatment in the hematology department. During his hospitalization in our hospital, he developed fever, abdominal pain, and lower back pain, and his face, penis, trunk, and limbs developed itchy rash. Subsequently, the patient had to undergo cardiopulmonary resuscitation because of sudden cardiac arrest, and be transferred to ICU for treatment. It was presumed that the cause is unknown severe sepsis. The patient’s condition quickly progressed to multiple organ failure, accompanied by liver, respiratory, and circulatory failure, and signs of disseminated intravascular coagulation. Unfortunately, the patient died after 8 h of active treatment. Finally, we collected all the evidence and concluded that the patient died of AA-PNH syndrome combined with poxzoster virus.

**Conclusion:**

AA-PNH syndrome patients treated with steroids and immunosuppressants are prone to various infections, considering that herpes virus infection with chickenpox and rash as the initial manifestations is characterized by rapid progress and often accompanied by serious complications. It is more difficult to distinguish it from AA-PNH syndrome with skin bleeding points. If it is not identified in time, it may delay the treatment opportunity, make the condition worse, and cause serious adverse prognosis. Therefore, clinicians need to pay attention to it.

## Introduction

Aplastic anemia (AA) is a syndrome characterized by bone marrow hypoplasia and failure and peripheral blood pancytopenia. At lower clonal abundance, paroxysmal nocturnal hemoglobinuria (PNH) cells can also be found in bone marrow failure disorders, such as AA, and in apparently healthy individuals (Parker, 2016; Hill et al., 2017). In the first-named condition referred to as AA-PNH, the disease is dominated by grossly reduced blood cell production rather than hemolysis (Young, 2018). Most patients need to use immunosuppressive therapy such as hormone and cyclosporine. The risk of concurrent infection increases sharply, and medical evaluation is required.

Varicella-zoster virus (VZV) is a kind of pathogenic human alphaherpesvirus (Zerboni et al., 2014). VZV usually causes primary infection in children. After a period of incubation, a widely distributed water blister appears, which is called varicella (also known as chickenpox) (Gershon et al., 2015); in individuals with normal immune function, only skin rash and flu-like symptoms, which are usually self-limiting. In special populations, such as immunocompromised individuals and pregnant women, varicella zoster virus infection may lead to serious or even fatal diseases, often due to bacterial superinfection (Tommasi and Breuer, 2022).

We report a case of 26-year-old man with a history of AA-PNH syndrome complicated with a typical separated VZV Infection, which was confirmed by clinical manifestations and laboratory evaluation. Although the outstanding condition of the patient in this case has been actively treated, the prognosis is poor.

## Case report

### Case presentation

Three months ago (July 17, 2022), a 26-year-old man was found with thrombocytopenia during physical examination, with a platelet count of 29 × 10^9^/L. At that time, the patient could see gingival bleeding when brushing his teeth. However, the patient did not pay enough attention. After 4 days, the patient went to the hematology department of our hospital. He completed blood routine test and bone marrow biopsy. A complete blood count disclosed pancytopenia (Hb: 9.8 g/dl; reticulocytes: 3.5 × 10^9^/L; leukocytes: 2.2 × 10^9^/L; neutrophils: 1.0 × 10^9^/L; and platelets: 30 × 10^9^/L). Blood marrow aspiration showed persistent severe hypoplasia with 25% cellularity, Megakaryocytes scattered occasionally. Cytofluorometric analysis was positive for the PNH clone (Babushok, 2021). AA-PNH syndrome was considered according to the test results. After communicating with the patient, the patient began to receive Traditional Chinese Medicine, hormone, and cyclosporine treatment. During the regular follow-up of the outpatient department, there was no significant fluctuation in various indicators.

On November 26, 2022, the patient went to the hospital again because of the persistent stabbing pain in the waist and back 2 days ago. Physical examination revealed that patient had discomfort and anemia appearance, unpalpable superficial lymph nodes, abdominal distension, abdominal muscle tension, tenderness, no shifting dullness, and limb edema. Eczema and swelling appeared on the glans and prepuce, accompanied by pruritus. Laboratory analyses showed Hb: 11.5 g/dl; reticulocytes: 3.1 × 10^9^/L; leukocytes: 2.7 × 10^9^/L; neutrophils: 1.8 × 10^9^/L; and platelets: 14 × 10^9^/L. C-reactive protein level was 0.4 mg/L. The liver function test showed that direct bilirubin (7.6 μmol/L), glutamic-oxalic transaminase (152 U/L), and glutamic-pyruvic transaminase (199 U/L) were slightly increased. Renal function test, urine examination, and coagulation profile were normal, and blood culture was sterile. Antinuclear antibodies titer was negative. Abdominal ultrasound only showed fatty liver. Renal ultrasound showed multiple strong echoes in the collection system of both kidneys, with the maximum diameter of about 0.4 cm. Cardiac ultrasound showed no obvious abnormality. Symptomatic treatment was carried out for the pain of the patient, and specialist consultation was invited for the perineal symptoms.

Days 3 after admission (November 28, 2022), the patient developed stubborn high fever with the highest temperature of 38.9°C, accompanied by rapid heart rate and tachypnea. At this time, the abdominal pain still persists and cannot be relieved. Abdominal computed tomography (CT) scan showed that fatty liver, gas accumulation, and expansion of colon, and more contents in intestinal cavity. Laboratory analyses showed Hb: 10.6 g/dl; leukocytes: 2.4 × 10^9^/L; neutrophils: 1.5 × 10^9^/L; and procalcitonin was 0.16 ng/ml. Considering that the patient had enterogenous infection and intestinal bleeding, he was empirically given anti-infection treatment.

**Figure 1:**
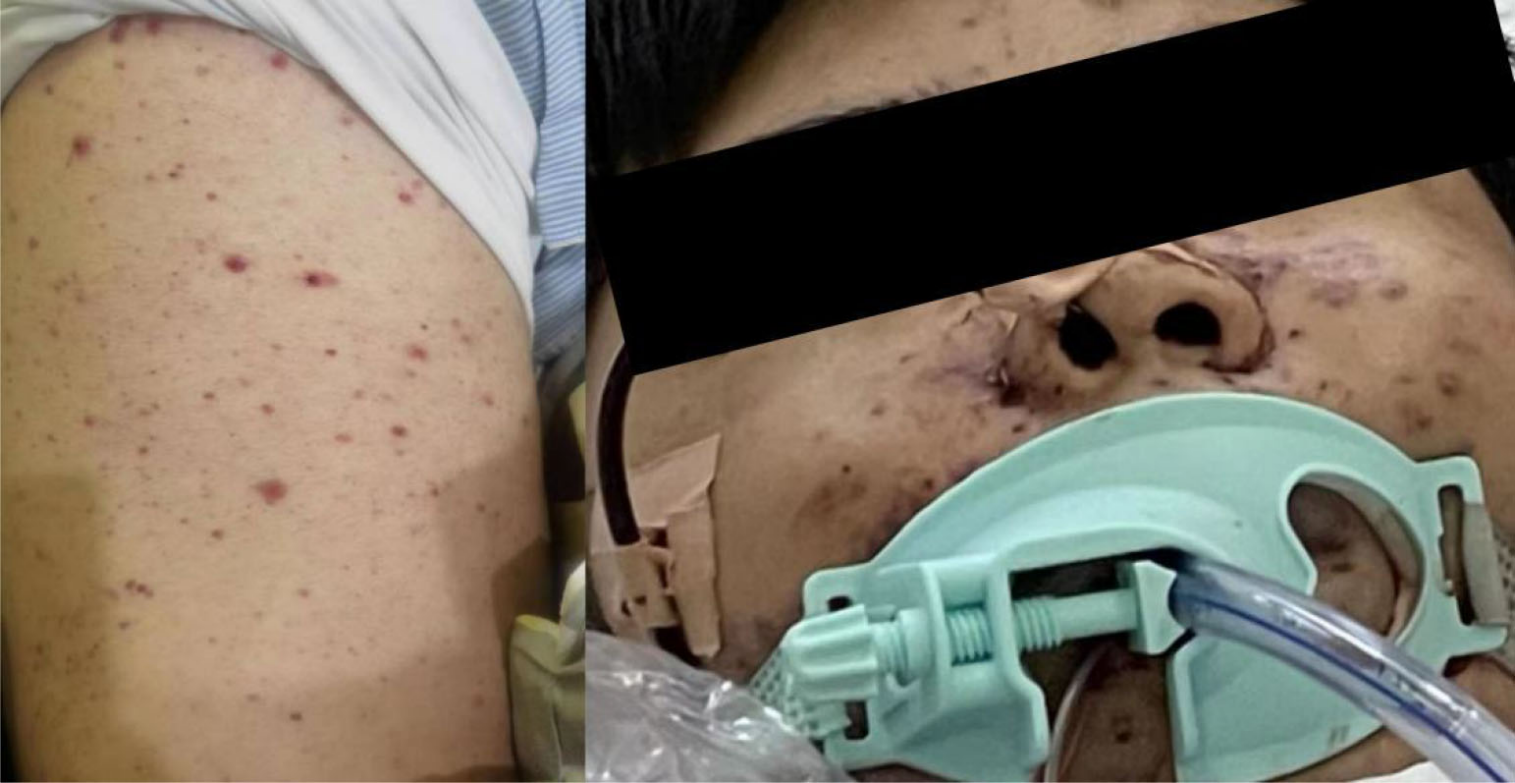
Representative clinical images from presentation showing a diffuse rash at different stages of development, including papules, vesicles, pustules, and crusted vesicles.

Days 4 after admission (November 29, 2022), at 9:30 a.m., the patient developed unconsciousness and difficulty breathing (blood pressure 90/40mmHg, pulse 39/min, respiratory rate 22/min, temperature 38.8°C, oxygen saturation 81% on nasal catheter oxygen inhalation 6L/min). After about 1 minute, the patient had cardiac arrest and was immediately rescued. After successful resuscitation, he was transferred to the intensive care unit for further treatment. At 11:30 a.m., the patient was in a coma condition, with ventilator-assisted breathing. His whole body had diffuse rashes at different stages of development, including vesicles, pustules, and crusted vesicles (**Figure 1**). As can be seen on the bedside electrocardiographic monitor: blood pressure was 87/53 mmHg (norepinephrine 11 μg/kg/min), HR (140–150)/min (atrial fibrillation), respiratory rate 15/min, temperature 35.2 °C; oxygen saturation cannot be measured (Fi O_2_ 100%). Laboratory analysis revealed Hb: 1.6 g/dl; leukocytes: 0.8 × 10^9^/L; neutrophils: 0.6 × 10^9^/L; and platelets: 9 × 10^9^/L (21U platelets were infused on November 28, 2022); procalcitonin was 0.26 ng/ml. Cardiac troponin I was 1.78 ng/ml. In addition, acute hepatic insufficiency (direct bilirubin 13.6 μmol/L, glutamic-oxalic transaminase 7360 U/L and glutamic-pyruvic transaminase 9780 U/L), acute renal insufficiency (creatinine 240.2 μmol/L) and blood coagulation disorder occurred (D-dimer > 80,000 μg/L, plasma protamine paracoagulation (3P) test was positive). Multiple organ failure caused by infection was first considered. We gave empirical anti-infection, blood product infusion, and life support treatment. However, it failed to save the life of the patient, and the clinical death was announced 8 h later. The day after the patient died, we had obtained the results of metagenomic next-generation sequencing (mNGS) (Han et al., 2019); only human herpesvirus type 3 can be seen in the blood of the patient, and no bacteria, fungi, and parasites were found, along with the appearance of lesions in the context of immunosuppression. A clinical diagnosis of disseminated VZV infection was made.

**Table 1** shows a timeline for our patient’s past medical history and follow-up visits as well as interventions.

**Table 1:** Timeline of patient’s medical history.

Hospital/out-patient visits date	Examination and chief complaint	Interventions
**2022/7/17**	White blood cell 3.1 *10^9^/L, Hemoglobin 101 g/L, Platelet 29 *10^9^/L. Bleeding gums.	Failure to pay attention.
**2022/7/21**	White blood cell 3.1 *10^9^/L, Hemoglobin 98g/L, Platelet 29 *10^9^/L, Reticulocyte 3.5%. Bleeding gums.	Planned to complete Bone marrow puncture biopsy.
**2022/8/4**	White blood cell 3.1 *10^9^/L, Hemoglobin 101g/L, Platelet 29 *10^9^/L, Reticulocyte 3.1%. Bone marrow puncture biopsy: bone marrow failure. PNH tested indicates positive.	TCM treatment (Patient requirement).
**2022/8/20**	White blood cell 3.1 *10^9^/L, Hemoglobin 104g/L, Platelet 29 *10^9^/L, Reticulocyte 3.2%.	The patient was treated with steroids.
**2022/8/29**	White blood cell 3.1 *10^9^/L, Hemoglobin 124g/L, Platelet 29 *10^9^/L, Reticulocyte 4.8%.	The patient began to receive Hetrombopag olamine for treatment.
**2022/11/4**	White blood cell 3.1 *10^9^/L, Hemoglobin 115g/L, Platelet 29 *10^9^/L, Reticulocyte 3.1%. Glutamic-pyruvic transaminase 88U/L.	The patient received cyclosporin A for treatment.
**2022/11/26**	White blood cell 3.1 *10^9^/L, Hemoglobin 116g/L, Platelet 29 *10^9^/L, Reticulocyte 3.3%. The patient had pain in the back and waist, accompanied by rash on the penis and glans. Glutamic-pyruvic transaminase 199U/L.	The patients were treated with paroxysmal pain and complete examination, and the treatment of steroid, cyclosporine A and hermopag olamine was stopped.
**2022/11/29**	Fever, skin rash and multiple organ failure.	Death.

PNH, paroxysmal nocturnal hemoglobinuria; TCM, traditional Chinese medicine.

## Discussion

This case reflects one important clinical learning point. Disseminated VZV infection can affect patients with AA-PNH syndrome who receive cyclosporine A and steroid. AA-PNH syndrome is a rare immune mediated hematopoietic disease with significant incidence rate and mortality. AA-PNH syndrome is treated according to AA principles (Young, 2018; Alashkar et al., 2020). AA is considered to be an immune-mediated disease. Immunosuppressive therapy and bone marrow transplantation are the main means of treatment for AA patients. As mentioned in our report, the patient was being treated with cyclosporine A and corticosteroids (Peslak et al., 2017; Young, 2018). We report a patient who was previously infected with VZV, but was re-infected with VZV during the immunosuppressive treatment of AA-PNH syndrome (Zawitkowska et al., 2020). The patient failed to be diagnosed and treated in time, and finally died. People with specific immunity to varicella zoster virus still have the chance to be infected again. With the aging of human host, the cell-mediated immunity to this virus in human body gradually decreases, and the virus reactivation becomes more frequent (Kennedy and Gershon, 2018; Laing et al., 2018).

It is generally believed that chickenpox is usually associated with mild diseases. Varicella zoster virus infection can be divided into primary infection or reactivation, the latter usually manifesting as rash and acute neuritis. Nevertheless, immunocompromised patients may have more severe and atypical manifestations, such as encephalitis, aseptic meningitis, pneumonia and hepatitis, along with disseminated visceral and cutaneous involvement (Vassia et al., 2020; Lenfant et al., 2022). Rash is the main clinical manifestation of our patients. Skin lesions often develop from papules to vesicles to scabs within a few days. However, severe rash takes longer to heal; accompanying symptoms include discomfort, fever, and fatigue, usually lasting for about a week. More serious cases may even be complicated with skin bacterial superinfection, encephalitis, and pneumonia. Adults and immunocompromised patients are more prone to severe infections than healthy children (Gershon et al., 2015; Nagel and Gilden, 2014). Severe or even fatal chickenpox, moreover, often occurs in patients with impaired immune function due to diseases or drugs, such as corticosteroids or cancer chemotherapy (Petrun et al., 2015; Lewis et al., 2017; Kennedy and Gershon, 2018; Loftus et al., 2019; Pannu and Manikandan, 2019).

The skin of patients with AA-PNH syndrome is prone to bleeding points, especially when the platelet level is significantly decreased. Our patient had skin “macula” at the time of last hospitalization, and our attending physician suspected it was a bleeding point after physical examination. However, it is possible that these “macula” are also mixed with some rashes caused by human herpes zoster virus. If we can identify and make a clear diagnosis at an early stage and give the patients antiviral treatment in time, the prognosis may not be known. It is worth considering that it is also unclear whether the abdominal pain of the patient is caused by early visceral disseminated varicella zoster virus infection, although abdominal CT scan did not find characteristic imaging manifestations (Furuto et al., 2019; Agatsuma et al., 2021). At present, clinical treatment found that most patients with varicella zoster virus infection had good prognosis after standard antiviral treatment (Andrei and Snoeck, 2021), and similar conclusions were also observed in immunocompromised patients (Andrei and Snoeck, 2021).

## Conclusion

AA-PNH syndrome patients treated with steroids and immunosuppressants are prone to various infections. Therefore, it is necessary to dynamically evaluate the benefits and risks of using steroid drugs or immunosuppressive drugs based on the patient’s situation in a timely manner. Considering that herpes virus infection with chickenpox and rash as the initial manifestations is characterized by rapid progress and often accompanied by serious complications. It is more difficult to distinguish it from AA-PNH syndrome with skin bleeding points. If it is not identified in time, it may delay the treatment opportunity, make the condition worse, and cause serious adverse prognosis. Therefore, clinicians need to pay attention to it.

## Data availability statement

The original contributions presented in the study are included in the article/supplementary material. Further inquiries can be directed to the corresponding author.

## Ethics statement

The studies involving human participants were reviewed and approved by Medical Ethics Committee of Hangzhou Red Cross Hospital. The patients/participants provided their written informed consent to participate in this study. Written informed consent was obtained from the individual(s) for the publication of any potentially identifiable images or data included in this article.

## Author contributions

JW and ZY: data curation, funding acquisition, and writing original draft. DR and ZL: investigation. KF, ZS, and ZL: resources and writing review and editing. All authors contributed to the article and approved the submitted version.
